# Metallothionein expression correlates with metastatic and proliferative potential in squamous cell carcinoma of the oesophagus

**DOI:** 10.1038/sj.bjc.6690753

**Published:** 1999-10

**Authors:** Y Hishikawa, T Koji, D K Dhar, S Kinugasa, M Yamaguchi, N Nagasue

**Affiliations:** Second Department of Surgery, Shimane Medical University, Izumo, 693-8501, Japan; Department of Histology and Cell Biology, Nagasaki University School of Medicine, Nagasaki, 852-8523, Japan

**Keywords:** metallothionein, in situ hybridization, oesophageal cancer, metastasis, cell proliferation

## Abstract

The goal of this study is to clarify whether the expression of metallothionein (MT) could affect the prognosis and the metastatic potential of squamous cell carcinoma (SCC) of the oesophagus. In paraffin-embedded specimens resected from 57 patients, MT mRNA and protein expressions were detected by in situ hybridization and immunohistochemistry respectively. The expression of MT was evaluated in respect of clinicopathologic variables and patients' survival. MT mRNA expression was significantly associated with the proportion of lymph node metastasis (71% in MT mRNA-positive tumours vs 42% in MT mRNA-negative tumours; *P* = 0.0343) and that of distant metastasis (29% in MT mRNA-positive tumours vs 5% in MT mRNA-negative tumours; *P* = 0.0452). In respect of MT protein expression, the frequency of distant metastasis was more common in MT-positive tumours than in MT-negative tumours (30% in MT-positive tumours vs 8% in MT-negative tumours; *P* = 0.0446). The survival rate of the patients with MT protein-negative tumours was significantly better than that of the patients with MT protein-positive tumours (*P* = 0.0340). There was a positive correlation between the expression of MT protein and that of proliferating cell nuclear antigen (*P* = 0.0018). Therefore, we conclude that MT expression, both at the mRNA and protein levels, may be a potential marker predicting metastatic and proliferative activities of oesophageal SCC. © 1999 Cancer Research Campaign


*British Journal of Cancer* (1999) **81**(4), 712–720
© 1999 Cancer Research Campaign
Article no. bjoc.1999.0753

### Metallothionein expression correlates with metastatic and proliferative potential in squamous cell carcinoma of the oesophagus

**Y Hishikawa[1,*], T Koji[2], DK Dhar[1], S Kinugasa[1], M Yamaguchi[1] and N Nagasue[1]**

[1]Second Department of Surgery, Shimane Medical University, Izumo 693-8501, Japan; [2]Department of Histology and Cell Biology, Nagasaki University School of Medicine, Nagasaki 852-8523, Japan

**Summary** The goal of this study is to clarify whether the expression of metallothionein (MT) could affect the prognosis and the metastatic potential of squamous cell carcinoma (SCC) of the oesophagus. In paraffin-embedded specimens resected from 57 patients, MT mRNA and protein expressions were detected by in situ hybridization and immunohistochemistry respectively. The expression of MT was evaluated in respect of clinicopathologic variables and patients' survival. MT mRNA expression was significantly associated with the proportion of lymph node metastasis (71% in MT mRNA-positive tumours vs 42% in MT mRNA-negative tumours; $P = 0.0343$) and that of distant metastasis (29% in MT mRNA-positive tumours vs 5% in MT mRNA-negative tumours; $P = 0.0452$). In respect of MT protein expression, the frequency of distant metastasis was more common in MT-positive tumours than in MT-negative tumours (30% in MT-positive tumours vs 8% in MT-negative tumours; $P = 0.0446$). The survival rate of the patients with MT protein-negative tumours was significantly better than that of the patients with MT protein-positive tumours ($P = 0.0340$). There was a positive correlation between the expression of MT protein and that of proliferating cell nuclear antigen ($P = 0.0018$). Therefore, we conclude that MT expression, both at the mRNA and protein levels, may be a potential marker predicting metastatic and proliferative activities of oesophageal SCC. © 1999 Cancer Research Campaign

**Keywords:** metallothionein; in situ hybridization; oesophageal cancer; metastasis; cell proliferation

Squamous cell carcinoma (SCC) of the oesophagus is well recognized for its aggressive biological behaviour, in respect of local infiltration and metastases to regional lymph nodes and distant organs (Orrigner, 1997). Extra-oesophageal tumour spread is present in 70% of cases at the time of diagnosis, and the 5-year survival is only 3% when lymph node metastases are present, compared with 42% in lymph node negative patients (Orrigner, 1997). Patients with distant metastasis also have a poor prognosis, with a 5-year survival rate of 5% or less (Mandard et al, 1984; Orrigner, 1997).

Metastasis is a multistep process involving complex associations of tumour cells with host cells or with matrix (Stracke and Liotta, 1995). Recently, genetic mechanisms underlying metastasis and pathogenesis of oesophageal SCC have been explored in several studies: int-2/hst-1 co-amplification correlated with high incidence of metastasis in distant organs (Kitagawa et al, 1991), loss of heterozygosity of the DCC gene associated with the degree of lymph node metastasis (Miyake et al, 1994), and mutation of the p53 gene in the pathogenesis (Hollstein et al, 1990; Meltzer et al, 1991) of SCC. However, there are only a limited number of reports on the prognostic indicators for invasion and metastasis of oesophageal SCC.

Metallothioneins (MTs) have been described in most vertebrate and invertebrate species as low molecular weight proteins. Overexpression of MT was described in a variety of human tumours, in relation to different stages of tumour development and progression (Jasani and Schmid, 1997). MT was considered as a potential prognostic marker in invasive ductal carcinoma of the breast by Schmid et al (1993) and others (Fresno et al, 1993; Goulding et al, 1995, Oyama et al, 1996). In skin carcinoma (Zelger et al, 1994), melanoma (Zelger et al, 1993), cervical carcinoma (Lim et al, 1996) and pancreatic carcinoma (Ohshio et al, 1996), MT overexpression appears to be predominantly associated with more advanced, highly malingant tumours. To the contrary, Öfner et al (1994) reported that MT overexpression in colorectal carcinoma was associated with better clinical outcome and tumour stages. Therefore, MT overexpression may be paradoxically associated in some tumours with better clinical outcome.

Tumour MT level also has been reported to correlate with resistance to anticancer reagents (Kelley et al, 1988; Kasahara et al, 1991; Chin et al, 1993; Kondo et al, 1995; Hishikawa et al, 1997). In oesophageal SCC, we already reported that MT protein expression might be a significant determinant of prognosis, in particular, associated with cisplatin resistance (Hishikawa et al, 1997). Patients with MT protein-negative tumours survived longer when treated with cisplatin than those with MT protein-positive tumours. However, the exact biological significance of the expression of MT protein and mRNA is largely unknown in the clinical setting of oesophageal SCC.

Therefore, in the present study, we have investigated the expressions of both MT mRNA and its protein in surgically resected oesophageal SCC by in situ hybridization and immunohistochemistry, respectively, and addressed whether MT expression could predict the prognosis and metastatic potential of oesophageal SCC

*Received 3 November 1998*
*Revised 6 February 1999*
*Accepted 20 April 1999*

*Correspondence to:* Y Hishikawa

*Present address: Department of Histology and Cell Biology, Nagasaki University School of Medicine, Nagasaki 852-8523, Japan without any pretreatment. Moreover, we have attempted to correlate the expression of MT with proliferating cell nuclear antigen (PCNA) expression, since in gastrointestinal tumours significant correlation have been noticed between proliferative activity and metastatic potential (Hall et al, 1990; Jain et al, 1991; Al-Sheneber et al, 1993; Hickey et al, 1994; Kinugasa et al, 1996).

### MATERIALS AND METHODS

### Patients

Between 1984 and 1994, 152 patients with oesophageal SCC were treated in the Second Department of Surgery, Shimane Medical University. Fifty-seven patients who were not treated with preoperative adjuvant therapies were selected for the analysis of MT mRNA by in situ hybridization, and that of MT protein and PCNA by immunohistochemistry. Ninety-five patients were excluded because of non-resectable tumours due to either disease that was too advanced or poor general conditions, death by surgical complications, preoperative adjuvant therapy for the tumour which might be induced MT, and early-stage diseases (carcinoma in situ). Thirty-six of the 57 patients received systemic chemotherapy and/or external radiotherapy postoperatively. Twenty-three patients of them were treated with cisplatin, 5-fluorouracil (5-Fu) and radiotherapy. Eight patients received cisplatin and 5-Fu without radiotherapy, and five patients received radiotherapy alone. Staging of the disease was based on the TNM staging system (UICC, 1992). Patients' clinicopathological characteristics are shown in Table 1. All patients or their next of kin provided informed consent for participation in the clinical study.

**Table 1** Clinicopathological characteristics of the 57 patients

| | Values |
|---|---|
| Sex (male/female) | 53/4 |
| Age, years (mean ± s.d.) | 63.7 ± 8.5 |
| Tumour location | |
| Upper/middle/lower | 9/27/21 |
| Tumour length (cm, mean ± s.d.) | 5.3 ± 2.5 |
| Histological differentiation | |
| Well/moderate | 46 |
| Poor | 11 |
| Stage (TNM) | |
| I/IIA/IIB/III/IV | 13/6/10/16/12 |
| Lymph node metastasis | |
| N0/N1 | 22/35 |
| Distant metastasis | |
| M0/M1 | 45/12 |
| Postoperative adjuvant therapy | |
| Cisplatin + 5-FU + radiation | 23 |
| Cisplatin + 5-FU | 8 |
| Radiation | 5 |
| No treatment | 21 |

### Tissue preparation

The surgical specimens were fixed in 10% buffered formalin solution and embedded in paraffin. The same paraffin-embedded block was used for in situ hybridization and immunohistochemistry. Five micron-thick sections were mounted on aminopropyltriethoxylsilane-coated glass slides.

### Oligo-DNAs

The oligo-DNA probe sequences of human MT for in situ hybridization were shown in Table 2. MT exists in different isoforms and most human MT genes, including the mainly functional ones (MT IA and MT IIA), are localized on chromosome 16 (Karin et al, 1984; Le Beau et al, 1985), and MT IIA is the major human MT gene, accounting for 50% of the MT mRNA expression. Then, to screen MT-positive cases, we used common oligonucleotide sequences shared among MT genes (93–98% homology) as a general MT probe set. The MT antisense oligo-DNA using thymine-thymine (T-T) dimerized DNA as a haptenic probe was selected complementary to the mRNA sequence coding for amino acids of the human MT IIA protein (Karin and Richards, 1982) and the MT sense oligo-DNA corresponded to the mRNA sequence (Table 2A). These two oligo-DNAs were added with three and two repeats of adenine-thymine-thymine (ATT) at the 5′ and 3′ ends, respectively, for the T-T dimers. We conducted a computer-assisted search (GenBank nucleic acid sequence database Release 102.0) of the above MT IIA oligo-DNA sequences (without the ATT repeats) and found 100% homology with MT IIA mRNA sequence and 93% with MT IA mRNA sequence. Therefore, to distinguish the MT IA and MT IIA mRNAs completely, we performed a second set of experiment with target sequences in MT IA and MT IIA mRNAs closer to the 3′ end of the message, where significant dissimilarities between mRNAs encoding different MT isoforms exist (Table 2B).

### Labelling and detection of oligo-DNAs

The MT oligo-DNAs were haptenized by introducing T-T dimers by UV irradiation as described in detail previously (Koji and Nakane, 1996). The optimal UV dose for these oligo-DNAs was 10 000 J $m^{-2}$ as determined by dot-blot hybridization, described below. Both the MT IA and MT IIA oligo-DNAs were labelled at their 5′- and 3′-end with digoxigenin (Dig)-11-dUTP by terminal deoxynucleotidyl transferase as described in detail previously (Koji and Brenner, 1993). The detection sensitivity of the T-T dimerized DNAs was examined immunohistochemically by using a HRP-linked mouse anti-T-T IgG (Kyowa Medex, Japan) and that of the Dig-labelled ones was done by using an anti-Dig-POD antibody (Boehringer Mannheim, Germany).

### Dot-blot hybridization

The procedures for dot-blot hybridization were described previously in detail (Yoshii et al, 1995; Koji and Nakane, 1996). Two microlitres of the sense oligo-DNA solution were placed on nitrocellulose membranes that had been pretreated with 20 × SSC (1 × SSC = 0.15 M sodium chloride and 0.015 M sodium citrate, pH 7.0) in a series of spots from 1 pg to 10 ng per spot. The membranes were hybridized at 42°C for 15–17 h with 2 μg $ml^{-1}$ T-T dimerized MT antisense probe or T-T dimerized MT sense probe. After successive washing, the membranes were immersed in a blocking solution for 1 h. The reaction with mouse anti-T-T IgG (diluted at 1:80 with the blocking solution) was performed for 3 h. After washing with phosphate-buffered saline (PBS), visualization of HRP sites was performed with diaminobenzidine–4 hydrochloric acid (DAB), $H_2O_2$, $Co^{2+}$, and $Ni^{2+}$ according to Adams (Adams, 1981).

© 1999 Cancer Research Campaign 

**Table 2** The oligo-DNA probe sequences of human MT[a] for in situ hybridization

| |
|---|
| (A) T-T dimerized MT probe |
| antisense oligo-DNA probe |
| ** **[b] ** ** ** |
| 5′ : TTATTA-CCT GGG CAC ACT TGG CAC AGC CCA CAG GGC AGC AGG AGC AGC AGC-ATTATTATT: 3′ |
| sense oligo-DNA probe |
| ** **[b] ** ** ** |
| 5′ : TTATTA-GCT GCT GCT CCT GCT GCC CTG TGG GCT GTG CCA AGT GTG CCC AGG-ATTATTATT: 3′ |
| (B) Digoxigenin-labeled MT probe |
| antisense oligo-DNA probe |
| MT IA 5′ : GTC ACT CTT TCT ATA TCT TCG AGC AGG GCT GTC CGG ACA TCA GGC : 3′ |
| MT IIA 5′: GTG GAA GTC GCG TTC TTT ACA TCT GGG AGC GGG GCT GTC CCA GCA : 3′ |
| sense oligo-DNA probe |
| MT IA 5′ : GCC TGA TGT CCG GAC AGC CCT GCT CGA AGA TAT AGA AAG AGT GAC : 3′ |
| MT IIA 5′ : TGC TGG GAC AGC CCC GCT CCC AGA TGT AAA GAA CGC GAC TTC CAC : 3′ |

[a]MT, metallothionein; T-T, thymine-thymine. [b]**, possible sites of T-T dimers.

### In situ hybridization

To estimate the amount of RNA retention level in paraffin sections, methyl green–pyronin Y staining (Shulte et al, 1992) was used before performing in situ hybridization in every run. The procedures for pretreatments were described previously in detail (Koji and Brenner, 1993; Koji and Nakane, 1996). Briefly, the sections were deparaffinized and rehydrated according to the standard procedures. These sections were treated with 0.2 N hydrochloric acid (RT, 20 min) and digested with 25 μg $ml^{-1}$ of proteinase K (37°C, 15 min). After post-fixation with 4% paraformaldehyde in PBS (5 min), the sections were immersed in 2 mg $ml^{-1}$ glycine in PBS (15 min) twice and kept in 40% deionized formamide in 4 × SSC until used for hybridization. Hybridization was carried out at 42°C for overnight with 2 μg $ml^{-1}$ T-T labelled oligo-DNA probes or with 2 μg $ml^{-1}$ Dig-labelled oligo-DNA probes dissolved in the hybridization medium. After repeated washings, the signals were detected by enzyme-immunohistochemistry as described above, without any counter-staining.

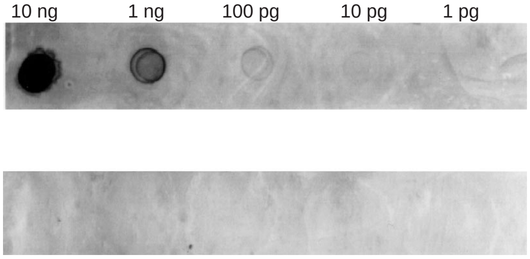

**Figure 1** Dot-blot hybridization. Various amounts (1 pg to 10 ng per spot) of MT sense oligo-DNA were hybridized with T-T-labelled MT antisense oligo-DNA (upper panel) or T-T labelled MT sense oligo-DNA (lower panel). Ten pg DNA was detected specifically.

### Control experiments

To confirm the specificity of MT mRNA signals, we conducted various types of control experiments concurrently with the test experiments. First, the sense probe was used as a negative control in every run. To evaluate the level of hybridizable RNAs in tissue sections, duplicate serial sections were used for 28S rRNA probe as a positive control in every case (Yoshii et al, 1995). Furthermore, some sections were hybridized with MT mRNA antisense probe in the presence of an excess amount of either homologous or heterologous unlabelled oligo-DNA to provide definitive evidence for the sequence specificity of the signal as described previously in detail (Koji and Brenner, 1993). To eliminate possible involvement of proteins and DNA in signal information, some sections were digested with RNase (100 mg $\mu l^{-1}$, 37°C, 1 h) before the post-fixation step (Koji and Brenner, 1993).

### Immunohistochemistry

Immunohistochemical staining was performed by the streptavidin–biotin (SAB) method using the SAB kit (Nichirei, Japan) according to the standard procedures. The monoclonal mouse anti-MT antibody (E9; Dako, USA) to an epitope of MT shared by its I and II isoforms of human, rat, horse MT was used at 1:100 dilution for 30 min at room temperature. This antibody has been used successfully to detect immunoreactive MT in formalin-fixed, paraffin-embedded tissues of human origin (Jasani and Elmes, 1991). To study the expression of PCNA, a mouse monoclonal antibody to PCNA (PC10; Dako, Denmark) was used at 1:100 dilution for 2 h at room temperature. AEC kit (Nichirei, Japan) for MT expression and DAB kit (Nichirei, Japan) for PCNA expression were used to visualize the sites of HRP. As positive control for MT staining, normal human colonic mucosa, which was already reported to express MT (Mulder et al, 1990; Steele et al, 1990) was used. To ensure consistency of PCNA staining, a known positive control oesophageal carcinoma was included in each run (Kinugasa et al, 1996). For the negative control, normal mouse IgG was used instead of the primary antibody.

### Statistical analysis

Mean values were compared with unpaired Student's *t*-test, and categorical variables were compared with Fisher's exact probability test. Actuarial survival was calculated by the Kaplan–Meier method and statistically evaluated by the log-rank test. Multivariate regression analysis was performed using step-wise multiple regression test among different clinical pathological factors. A *P*-value of less than 0.05 was considered statistically significant. All the analyses were performed with a statistical software package (Stat View, ver. 4.51; Abacus Concepts, Berkely, CA, USA).

© 1999 Cancer Research Campaign

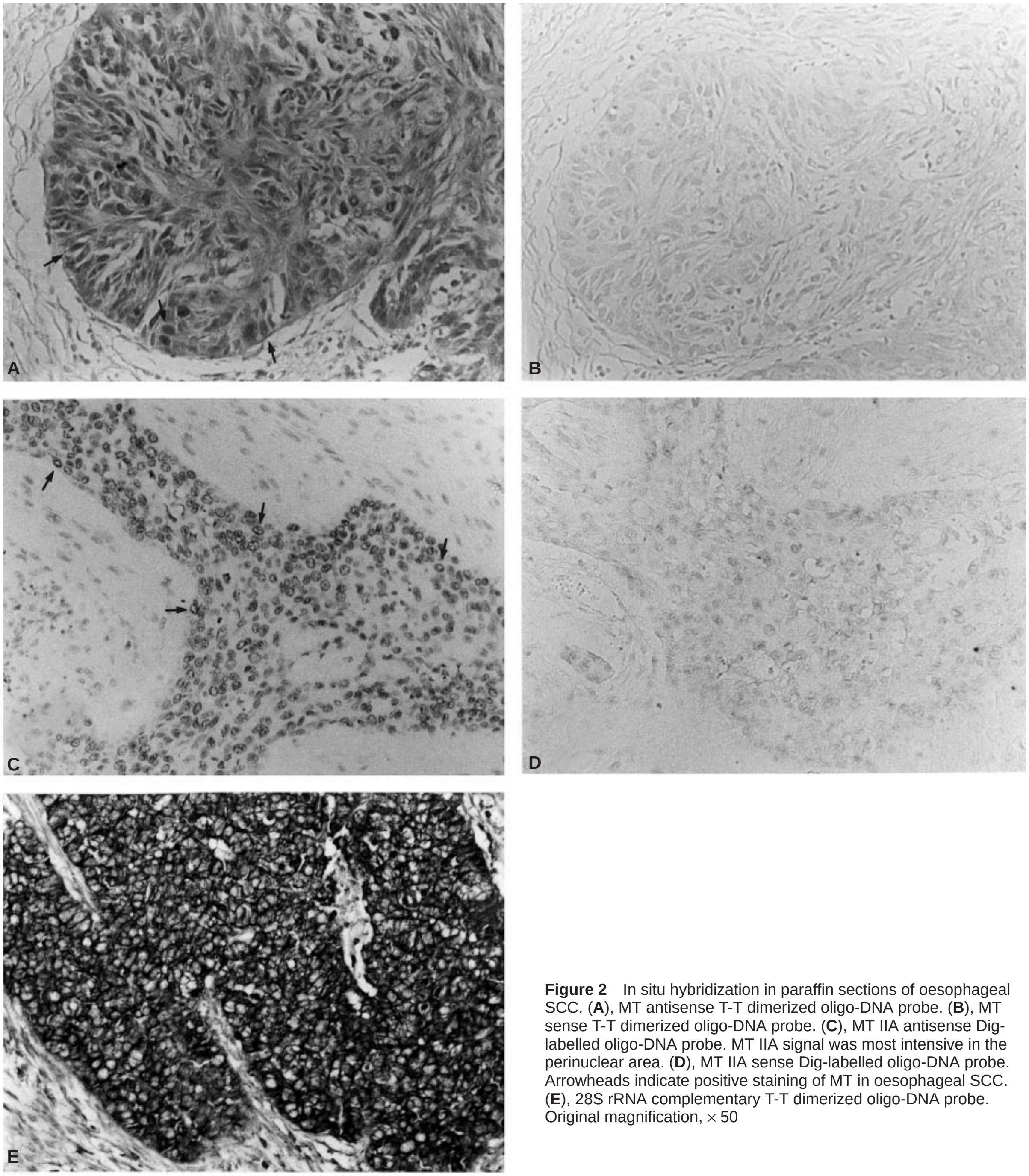

**Figure 2** In situ hybridization in paraffin sections of oesophageal SCC. (**A**), MT antisense T-T dimerized oligo-DNA probe. (**B**), MT sense T-T dimerized oligo-DNA probe. (**C**), MT IIA antisense Dig-labelled oligo-DNA probe. MT IIA signal was most intensive in the perinuclear area. (**D**), MT IIA sense Dig-labelled oligo-DNA probe. Arrowheads indicate positive staining of MT in oesophageal SCC. (**E**), 28S rRNA complementary T-T dimerized oligo-DNA probe. Original magnification, × 50

### RESULTS

### Dot-blot hybridization with T-T dimerized oligo-DNA

When T-T dimerized MT antisense probe was hybridized with unhaptenized MT sense oligo-DNA fixed on a nitrocellulose filter, 10 pg of oligo-DNA was detectable (Figure 1). However, with the sense probe no staining was found. For all probes used in the present study, similar results were obtained (data not shown). These findings indicated that the antisense probes we synthesized were specific and had adequate sensitivity to be useful for in situ hybridization studies.

### Localization of MT mRNA by in situ hybridization

The staining intensities for MT mRNA were graded as negative, no staining or positive, weak and intense staining, as compared to that with sense probe. MT mRNA expression for using T-T dimer as a general MT probe set was detected in 38 of the 57 patients (Figure 2A). As a negative control, when a section was hybridized with T-T-dimerized MT sense probe, no signal were observed (Figure 2B). Therefore, as the next step, in situ hybridization with specific probes to MT IA and MT IIA was performed to distinguish between the MT IA and MT IIA mRNAs in the MT mRNA-positive cases. Ten of the 38 patients with MT mRNA-positive staining

© 1999 Cancer Research Campaign

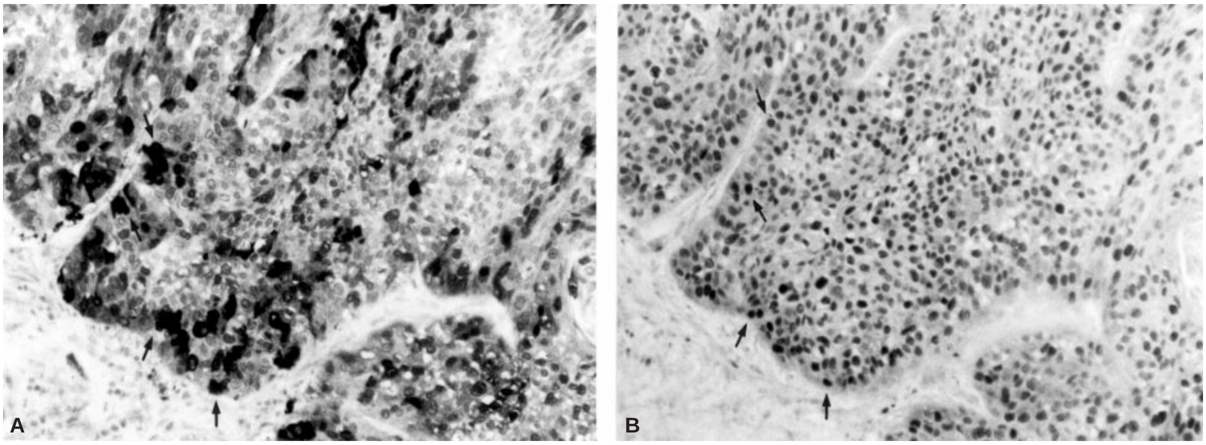

**Figure 3** Immunohistochemical staining of (**A**) MT protein and (**B**) PCNA in paraffin sections of oesophageal SCC. Arrows indicate positive staining both MT protein and PCNA expressions in the same area of the invading edge of the tumour. Original magnification, × 50

were randomly selected for the experiment. MT IIA mRNA expression was detected in all ten cases (Figure 2C), but MT IA mRNA expression could not be detected. As a negative control, no staining was found with Dig-labelled MT IIA sense probe in an adjacent section (Figure 2D). 28S rRNA probe used as a positive control gave an intense staining in cancer cells (Figure 2E).

### Localization of MT protein by immunohistochemistry

Expression of MT protein using immunohistochemistry was estimated visually as negative, very weak (scattered positive cells were less than 5% in the carcinomatous tissue), or positive (stained cells more than 5%). In the following analyses, patients with very weakly stained sections were categorized as the negative group. MT protein was localized in nuclei and/or cytoplasm (Figure 3A). MT protein expression was detected in 33 of the 57 patients.

### Inconsistency of MT mRNA and protein expression

MT mRNA-positive cases were 38 of the 57 patients by in situ hybridization, and MT protein-positive cases were 33 by immunohistochemistry. The inconsistency of MT mRNA and protein expression was shown in Table 3. Twenty-five of the 57 tumours were positive, and 11 tumours were negative for MT expression at both mRNA and protein levels. In 13 tumours MT was detectable only at the mRNA level, whereas in the remaining eight tumours it was detected only at the protein level.

### Correlation of MT expression with metastasis and patient’s survival

Comparisons were made between MT mRNA and MT protein expressions and the following variables: age, sex, tumour length, tumour location, histological differentiation, stage (TNM), lymph node metastasis (N0; no lymph node metastasis, or N1; regional lymph node metastasis) and distant metastasis (M0; no distant metastasis, or M1; distant metastasis). Only significant factors of this analysis have been shown in Table 4. The proportion of lymph node metastasis was 71% in patients with MT mRNA-positive tumours and 42% in those with MT mRNA-negative tumours ($P$ = 0.0343).

**Table 3** Inconsistency of MT mRNA and protein expression

| No. of patients | MT expression (mRNA, protein) |
|---|---|
| 25 | (positive, positive) |
| 13 | (positive, negative) |
| 8 | (negative, positive) |
| 11 | (negative, negative) |

The proportion of distant metastasis was 29% in MT mRNA-positive patients and 5% in MT mRNA-negative patients ($P$ = 0.0452). Similarly, in respect of MT protein expression, the frequency of distant metastasis was more common in MT protein-positive tumours than in MT protein-negative tumours (30% versus 8%; $P$ = 0.0446).

The patients with MT protein-negative tumours survived better than those with MT protein-positive tumours ($P$ = 0.0340; 95% confidence interval for MT negative, 0.522–0.911, and for MT positive, 0.161–0.518) (Figure 4). Survival rate between the patients with MT mRNA-negative and -positive tumours, however, was not significantly different (data not shown).

### MT expression and its relation to cell proliferation assessed by PCNA immunostaining

Fifty of the 57 patients were selected for the evaluation of PCNA staining because of the adequate nuclear form and low background. The staining was judged as positive when cancer cell nuclei were stained in equal intensity to the parabasal cells in normal oesophageal epithelium (Figure 3B). On average, we counted at least 1000 nuclei in randomly selected ten high power fields in each specimen. PCNA staining varied within a range of 0–48% with a mean value ± s.d. (16.7 ± 12.0), so the lower cut-off point for PCNA positivity was 5% of the ratio of positive nuclei.

In 28 of the 50 cases, which were satisfactorily evaluated for both PCNA and MT protein expressions, 93% of cases with both PCNA and MT protein expressions were mainly observed at the invasive edge of the tumour, as shown in serial sections stained by anti-MT and anti-PCNA (Figure 3A and 3B). Correlation of MT and PCNA

 © 1999 Cancer Research Campaign

**Table 4** Significant factors of clinicopathological characteristics of MT[a] mRNA and MT protein expression

| Factors | MT mRNA | | | MT protein | | |
|---|---|---|---|---|---|---|
| | Negative (*n* = 19) | Positive (*n* = 38) | *P*-value | Negative (*n* = 24) | Positive (*n* = 33) | *P*-value[b] |
| Lymph node metastasis (N) | | | | | | |
| N0 | 11 | 11 | 0.0343 | 12 | 10 | 0.1315 |
| N1 | 8 | 27 | | 12 | 23 | |
| Distant metastasis (M) | | | | | | |
| M0 | 18 | 27 | 0.0452 | 22 | 23 | 0.0446 |
| M1 | 1 | 11 | | 2 | 10 | |

[a]MT, metallothionein; N0, no regional node metastasis; N1, regional node metastasis; M0, no distant metastasis; M1, distant metastasis. [b]Fisher's exact probability test.

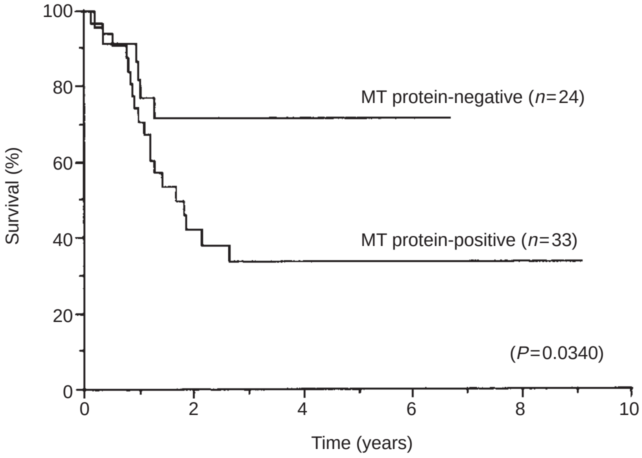

**Figure 4** The survival curves of MT protein-negative and -positive tumours. The patients with MT protein-negative tumours survived better than those with MT protein-positive tumours (*P* = 0.0340; 95% confidence interval for MT-negative, 0.522–0.911, and for MT-positive, 0.161–0.518)

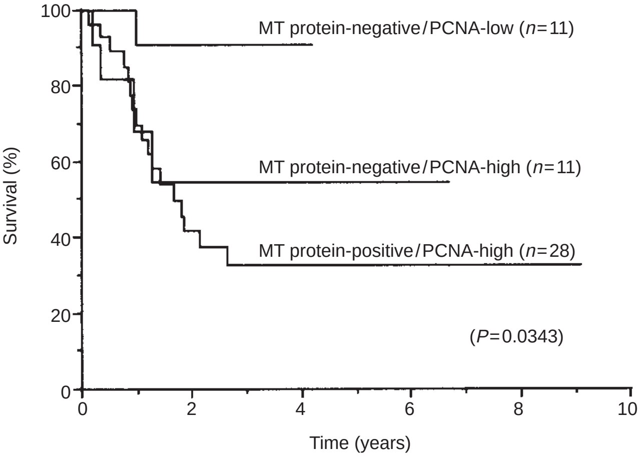

**Figure 5** The survival curves of combined MT protein and PCNA expression. The patients with MT protein-negative/PCNA-low tumours survived better than those with MT protein-negative/PCNA-high and MT protein-positive/PCNA-high tumours (*P* = 0.0343; 95% confidence interval for MT-negative/PCNA-low, 0.739–1.079, for MT-negative/PCNA-high, 0.201–0.889, and for MT-positive/PCNA-high, 0.140–0.514)

expression is shown in Table 5. A higher percentage rate of nuclear staining for PCNA antigen was observed in MT protein-positive tumours than in those -negative tumours ($P$ = 0.0018). All MT protein-positive tumours were in the PCNA-high tumours group ($P < 0.0001$). On the other hand, the rate of PCNA expression was not different between lymph node metastasis (N0 = 14.7 ± 13.9% versus N1 = 18.1 ± 10.6%; $P$ = 0.3331) and distant metastasis (M0 = 15.1 ± 12.3% versus M1 = 23.0 ± 8.3%; $P$ = 0.0590).

Survival rates according to the expression of MT protein and PCNA are shown in Figure 5. The patients with MT protein-negative/PCNA-low tumours survived better than those with MT protein-negative/PCNA-high and MT protein-positive/PCNA-high tumours ($P$ = 0.0343; 95% confidence interval for MT-negative/PCNA low, 0.739–1.079, for MT-negative/PCNA-high, 0.201–0.889, and for MT-positive/PCNA-high, 0.140–0.514).

**Table 5** Correlation between MT[a] and PCNA expression

| | MT mRNA | | | MT protein | | |
|---|---|---|---|---|---|---|
| | Negative (*n* = 17) | Positive (*n* = 33) | *P*-value | Negative (*n* = 22) | Positive (*n* = 28) | *P*-value |
| PCNA (%, mean ± SD) | 18.5 ± 19.8 | 16.7 ± 10.6 | 0.6777 | 10.9 ± 11.5 | 21.2 ± 10.5 | 0.0018[b] |
| Evaluation of PCNA | | | | | | |
| Low | 5 | 6 | 0.3638 | 11 | 0 | <0.0001[c] |
| High | 12 | 27 | | 11 | 28 | |

[a]MT, metallothionein; PCNA, proliferating cell nuclear antigen. [b]Student's *t*-test. [c]Fisher's exact probability test.

### Multivariate analysis

Multivariate analysis was conducted among the different clinicopathological variables by using step-wise multiple regression test. Tumour size (> 6 cm or 6 cm; ≥ $P < 0.0001$), lymph node metastasis (N0 or N1; $P = 0.0133$), and distant metastasis (M0 or M1; $P = 0.0353$) were independent prognostic factors for overall survival. Patient's age, gender, histological differentiation, postoperative therapies (treated or not; cisplatin, 5-FU, radiation), and staining intensity of MT protein and that of PCNA were not found to be an independent predictor of prognosis.

### DISCUSSION

In the present study, we have investigated the expression of MT mRNA and its protein in oesophageal SCC by non-radioactive in situ hybridization and immunohistochemistry respectively. As a result, we have found that MT expression is correlated with high incidence of tumour metastasis in patients with oesophageal SCC. In fact, we previously reported that the expression of MT protein in SCC of the oesophagus could be a significant determinant of poor prognosis in patients with cisplatin (Hishikawa et al, 1997). In the previous study, however, we could not avoid the possible effect of preoperative adjuvant therapies on the expression of MT. Therefore, we confirmed here the previous findings in the patients without preoperative treatment and also extended our analysis of MT expression to its transcript level.

The human MT mRNAs for MT I and MT II are highly homologous and substantial cross-hybridization with transcripts of other MT isoforms can be expected with the conventional probes. First, we searched the positivity of MT mRNA expression using first set oligo-DNA probe (T-T dimered one), and the expression of MT mRNA in oesophageal SCC was found in 66.6% of our patients. Therefore, we chose more specific target sequences for MT IA and MT IIA mRNAs closer to the 3′ end of the message further to confirm the specific expression of MT IA and MT IIA mRNAs. About ten MT mRNA-positive cases were randomly selected. MT IIA mRNA expression was found in all cases and MT IA mRNA expression was not detectable in any of these cases. Therefore, it is suggested that the expression of MT mRNA, mainly MT IIA mRNA, might be associated with the genetic mechanisms underlying metastasis in oesophageal SCC.

MT mRNA expression was positively correlated with metastasis to regional lymph nodes, and both MT mRNA and its protein levels were elevated in cases with distant metastases. Regarding the induction of MT gene expression, it is well known that MT gene contains AP-1 site in the regulatory region (Lee et al, 1987) and the transcription of MT gene is activated in part by this signal-transduction pathway (Skroch et al, 1991). Hu et al (1994) have reported that c-fos may play an important regulatory role in the invasive behaviour of malignant tumours. This oncogene product forms heterodimers with c-jun protein, and the resulting complex binds AP-1 sites of DNA and stimulates specific gene transcription through these elements (Chiu et al, 1988; Ransone and Verma, 1990), implying the possible relationship between MT gene expression and metastatic activity.

The available information on the character and consequences of MT overexpression associated with human cancer is presently limited (Jasani and Schmid, 1997), therefore, the actual role of MT related to invasiveness and metastasis is largely unknown in the clinical setting. In a mouse melanoma cell invasion model, it was shown that heavy metal-induced MT serves as a host-derived factor for cancer cell metastasis (Haga et al, 1996*a*). Recently, it was noted that MT enhances gelatinase A activation and induces cancer metastasis in vivo (Haga et al, 1996*b*). The significant correlation of MT expression with frequent occurrence of lymph node metastasis in this study indicates that MT might be a host-related factor for cancer metastasis. Furthermore, in MCF-7 cells, human breast cancer cell line, transfected with MT antisense oligonucleotide, the cell growth was remarkably inhibited and their apoptosis was induced (Abdel-Mageed and Agrawal, 1997). In the future, it could be possible to regulate the metastatic potential of the oesophageal SCC by antisense MT gene therapy.

The results obtained for MT protein and mRNA expression seemed to be inconsistent. Thirty-six of the 57 patients expressed MT equally at the mRNA and protein level, whereas 21 patients showed discrepancy. The controversial results in the 21 patients could be explained in the following ways:

1. there are differences in their half-lives or synthetic rates of mRNA and protein, so that the level of MT mRNA expression is not always directly proportional to that of MT protein (Skroch et al, 1991; Tohyama et al, 1993)
2. MT expression may be regulated at the post-transcriptional level.

This post-transcriptional control of MT expression has not been considered in the previous studies and needs further evaluation of the regulatory mechanisms at this level.

PCNA has been considered to be a useful marker of proliferating activity in some tumours (Hall et al, 1990; Jain et al, 1991; Al-Sheneber et al, 1993), including oesophageal SCC (Hickey et al, 1994; Kinugasa et al, 1996), and appears at the late G1/S boundary in the cell cycle. Nagel and Vallee (1995) reported that the level of MT during the mitotic cell cycle of human colon cancer cells reached its maximum near the G1/S boundary of the cell cycle, around the onset of DNA synthesis, raising a physiological role for MT in cell proliferation. Similarly, MT protein expression singly or in combination with PCNA expression was significantly associated with poor prognosis in oesophageal SCC in the univariate analysis, while, in the step-wise multiple regression test, neither MT expression nor PCNA could be an independent prognostic factor in oesophageal SCC. Therefore, in the clinical setting, the expression of MT protein might be a useful marker to identify cell proliferation in combination with PCNA. However, the predictability of prognosis by MT expression might be associated with other cell cycle regulatory genes, like p53 (Zheng et al, 1996).

In conclusion, the expression of MT protein and/or mRNA was positively correlated with tumour metastatic activity. The expression of MT protein was also correlated with tumour proliferating activity in the current study. These results indicate that MT mRNA and its protein may be used as potential markers, which permit us to predict the metastatic and proliferative activities in oesophageal SCC.

### ACKNOWLEDGEMENTS

We would like to thank Dr Paul K Nakane (Microcide Pharmaceuticals) for valuable advice.

### REFERENCES

Abdel-Mageed A and Agrawal KC (1997) Antisense down-regulation of metallothionein induces growth arrest and apoptosis in human breast carcinoma cells. *Cancer Gene Ther* **4**: 199–207

© 1999 Cancer Research Campaign

Adams JC (1981) Heavy metal intensification of DAB-based HRP reaction products. *J Histochem Cytochem* **29**: 775

Al-Sheneber IF, Shibata HR, Sampalis J and Jothy S (1993) Prognostic significance of proliferating cell nuclear antigen expression in colorectal cancer. *Cancer* **71**: 1954–1959

Chin JL, Banerjee D, Kadhim SA, Kontozoglou TE, Chauvin PJ and Cherian MG (1993) Metallothionein in testicular germ cell tumors and drug resistance. *Cancer* **72**: 3029–3035

Chiu R, Boyle WJ, Meek J, Smeal T, Hunter T and Karin M (1988) The c-fos protein interacts with jun/AP-1 to stimulate transcription of AP-1 responsive genes. *Cell* **54**: 541–552

Fresno M, Wu W, Rodriguez JM and Nadji M (1993) Localization of metallothionein in breast carcinomas. An immunohistochemical study. *Virchows Arch A* **423**: 215–219

Goulding H, Jasani B, Pereira H, Reid A, Galea M, Bell JA, Elston CW, Robertson JF, Blamey RW, Nicholson RA, Schmid KW and Ellis IO (1995) Metallothionein expression in human breast cancer. *Br J Cancer* **72**: 968–972

Haga A, Nagase H, Kito H and Sato T (1996*a*) Enhanced invasiveness of tumour cells after host exposure to heavy metals. *Eur J Cancer* **32A**: 2342–2347

Haga A, Nagase H, Kito H and Sato T (1996*b*) Effect of metallothioneins on transformation of gelatinase A from human fibroblast WI-38 cells. *Cancer Lett* **105**: 175–180

Hall PA, Levison DA, Woods AL, Yu CCW, Kellock DB, Watkins JA, Barnes DM, Gillett CE, Camplejohn R, Dover R, Waseem NH and Lane DP (1990) Proliferating cell nuclear antigen (PCNA) immunolocalization in paraffin sections: an index of cell proliferation with evidence of deregulated expression in some neoplasms. *J Pathol* **162**: 285–294

Hickey K, Grehan D, Reid IM, O'Briain S, Walsh TN and Hennessy TPJ (1994) Expression of epidermal growth factor receptor and proliferating cell nuclear antigen predicts response of esophageal squamous cell carcinoma to chemoradiotherapy. *Cancer* **74**: 1693–1698

Hishikawa Y, Abe S, Kinugasa S, Yoshimura H, Monden N, Igarashi M, Tachibana M and Nagasue N (1997) Overexpression of metallothionein correlates with chemoresistance to cisplatin and prognosis in esophageal cancer. *Oncology* **54**: 342–347

Hollstein MC, Metcalf RA, Welsh JA, Montesano R and Harris C C (1990) Frequent mutation of the p53 gene in human esophageal cancer. *Proc Natl Acad Sci USA* **87**: 9958–9961

Hu E, Mueller E, Oliviero S, Papaioannou VE, Johnson R and Spiegelman BM (1994) Targeted disruption of the c-fos gene demonstrates c-fos-dependent and -independent pathways for gene expression stimulated by growth factors or oncogenes. *EMBO J* **13**: 3094–3103

Jain S, Filipe MI, Hall PA, Waseem N, Lane DP and Levison DA (1991) Prognostic value of proliferating cell nuclear antigen in gastric carcinoma. *J Clin Pathol* **44**: 655–659

Jasani B and Schmid KW (1997) Significance of metallothionein overexpression in human tumours. *Histopathology* **31**: 211–214

Jasani B and Elmes ME (1991) Immunohistochemical detection of metallothionein. In: *Methods in Enzymology Metallobiochemistry, Part B, Metallothionein and Related Molecules*, Riordan JF and Vallee BL (eds), pp. 95–107. Academic Press: San Diego

Karin M and Richards RI (1982) Human metallothionein genes – primary structure of the metallothionein-II gene and a related processed gene. *Nature* **299**: 797–802

Karin M, Eddy RL, Henry WM, Haley LL, Byers MG and Shows TB (1984) Human metallothionein genes are clustered on chromosome 16. *Proc Natl Acad Sci USA* **81**: 5494–5498

Kasahara K, Fujiwara Y, Nishio K, Ohmori T, Sugimoto Y, Komiya K, Matsuda T and Saijo N (1991) Metallothionein content correlates with the sensitivity of human small cell lung cancer cell lines to cisplatin. *Cancer Res* **51**: 3237–3242

Kelley SL, Basu A, Teicher BA, Hacker MP, Hamer DH and Lazo JS (1988) Overexpression of metallothionein confers resistance to anticancer drugs. *Science* **241**: 1813–1815

Kinugasa S, Tachibana M, Hishikawa Y, Abe S, Yoshimura H, Monden M, Dhar DK and Nagasue N (1996) Prognostic significance of proliferating cell nuclear antigen (PCNA) in squamous cell carcinoma of the esophagus. *Jpn J Clin Oncol* **26**: 405–410

Kitagawa Y, Ueda M, Ando N, Shinozawa Y, Shimizu N and Abe O (1991) Significance of int-2/hst-1 coamplification as a prognostic factor in patients with esophageal squamous carcinoma. *Cancer Res* **51**: 1504–1508

Koji T and Brenner RM (1993) Localization of estrogen receptor messenger ribonucleic acid in rhesus monkey uterus by nonradioactive in situ hybridization with digoxigenin-labeled oligodeoxynucleotides. *Endocrinology* **132**: 382–392

Koji T and Nakane PK (1996) Recent advances in molecular histochemical techniques: in situ hybridization and southwestern histochemistry. *J Electron Microsc* **45**: 119–127

Kondo Y, Kuo S, Watkins SC and Lazo JS (1995) Metallothionein localization and cisplatin resistance in human hormone-independent prostatic tumor cell lines. *Cancer Res* **55**: 474–477

Le Beau MM, Diaz MO, Karin M and Rowley JD (1985) Metallothionein gene cluster is split by chromosome 16 rearrangements in myelomocytic leukaemia. *Nature* **313**: 709–711

Lee W, Mitchell P and Tjian R (1987) Purified transcription factor AP-1 interacts with TPA-inducible enhancer elements. *Cell* **49**: 741–752

Lim K, Evans A, Adams M, Fish R, Dhundee J, Dallimore N and Jasani B (1996) Association of immunohistochemically detectable metallothionein (IDMT) expression with malignant transformation in cervical neoplasia. *J Pathol* **178**: 48A

Mandard AM, Marnay J, Gignoux M, Segol P, Blanc L, Ollivier JM, Borel B and Mandard JC (1984) Cancer of the esophagus and associated legions: detailed pathologic study of 100 esophagectomy specimens. *Hum Pathol* **15**: 660–669

Meltzer SJ, Yin J, Huang Y, McDaniel TK, Newkirk C, Iseri O, Vogelstein B and Resau JH (1991) Reduction to homozygosity involving p53 in esophageal cancers demonstrated by polymerase chain reaction. *Proc Natl Acad Sci USA* **88**: 4976–4980

Miyake S, Nagai K, Yoshino K, Oto M, Endo M and Yuasa Y (1994) Point mutations and allelic deletion of tumor suppressor gene DCC in human esophageal squamous cell carcinomas and their relation to metastasis. *Cancer Res* **54**: 3007–3010

Mulder TPJ, Verspaget HW, Janssens AR, De Bruin PAF, Griffioen G and Lamers CBHW (1990) Neoplasia-related changes of two copper (Cu)/Zinc (Zn) proteins in the human colon. *Free Radical Bio Med* **10**: 501–506

Nagel WW and Vallee B (1995) Cell cycle regulation of metallothionein in human colonic cancer cells. *Proc Natl Acad Sci USA* **92**: 579–583

Öfner D, Maier H, Riedmann B, Bammer T, Rumer A, Winde G, Böcker W, Jasani B and Schmid KW (1994) Immunohistochemical metallothionein expression in colorectal adenocarcinoma: correlation with tumour stage and patient survival. *Virchows Arch* **425**: 491–497

Ohshio G, Imamura T, Okada N, Wang ZH, Yamaki K, Kyogoku T, Suwa H, Yamabe H and Imamura M (1996) Immunohistochemical study of metallothionein in pancreatic carcinomas. *J Cancer Res Clin* **122**: 351–355

Orrigner MB (1997) Tumors of the esophagus. In: *Textbook of Surgery*, Sabiston DC Jr (ed), pp. 744–758. WB Saunders: Philadelphia

Oyama T, Takei H, Hikino T, Iino Y and Nakajima T (1996) Immunohistochemical expression of metallothionein in invasive breast cancer in relation to proliferative activity, histology and prognosis. *Oncology* **53**: 112–117

Ransone LJ and Verma IM (1990) Nuclear proto-oncogenes fos and jun. *Annu Rev Cell Biol* **6**: 539–557

Schmid KW, Ellis IO, Gee JMW, Darke BM, Lees WE, Kay J, Cryer A, Stark JM, Hittmair A, Öfner D, Dünser M, Margreiter R, Daxenbichler G, Nicholson RI, Bier B, Böcker W and Jasani B (1993) Presence and possible significance of immunocytochemically demonstrable metallothionein over-expression in primary invasive ductal carcinoma of the breast. *Virchows Arch A* **422**: 153–159

Shulte EKW, Lyon HO and Hoyer PE (1992) Simultaneous quantification of DNA and RNA in tissue sections. A comparative analysis of the methyl green-pyronin technique with the gallocyanin chromalum and Feulgen procedures using image cytometry. *Histochem J* **24**: 305–310

Skroch P, Inouye C and Karin M (1991) Regulation of human and yeast metallothionein gene transcription by heavy metal ions. In: *Metallothionein in Biology and Medicine*, Klaassen CD and Suzuki KT (eds), pp. 61–74. CRC Press: Boca Raton, Florida

Steele GD Jr, Davis S, Yow H, Wong JM, Rivers EN, Summerhayes IC, Ravikumar TS and Chen LB (1990) Alterations of gene expression in human colorectal cancer. *Arch Surg* **25**: 493–497

Stracke ML and Liotta LA (1995) Molecular mechanisms of tumor cell metastasis. In: *The Molecular Basis of Cancer*, Mendelsohn J, Howley PM, Israel MA and Liotta LA (eds), pp. 233–247. WB Saunders: Philadelphia

Tohyama C, Suzuki JS, Hemelraad J, Nishimura N and Nishimura H (1993) Induction of metallothionein and its localization in the nucleus of rat hepatocytes after partial hepatectomy. *Hepatology* **18**: 1193–1201

Yoshii A, Koji T, Ohsawa N and Nakane PK (1995) In situ localization of ribosomal RNAs is a reliable reference for hybridizable RNA in tissue sections. *J Histochem Cytochem* **43**: 321–328

Zelger B, Hittmair A, Schir M, Öfner C, Öfner D, Fritsch PO, Böcker W, Jasani B and Schmid KW (1993) Immunohistochemically demonstrated metallothionein expression in malignant melanoma. *Histopathology* **23**: 257–264

© 1999 Cancer Research Campaign

Zelger B, Sidoroff A, Hopfl R, Öfner D, Jasani B and Schmid KW (1994) Metallothionein expression in nonmelanoma skin cancer. *Appl Immunohistochem* **2**: 254–260

Zheng H, Liu J, Choo KH, Michalska AE and Klaassen CD (1996) Metallothionein-I and -II knock-out mice are sensitive to cadmium-induced liver mRNA expression of c-jun and p53. *Toxicol Appl Pharm* **136**: 229–235

 *© 1999 Cancer Research Campaign*

## References

[PDF_00604] Abdel-Mageed A., Agrawal K. C. (1997). Antisense down-regulation of metallothionein induces growth arrest and apoptosis in human breast carcinoma cells.. Cancer Gene Ther.

[PDF_00607] Adams J. C. (1981). Heavy metal intensification of DAB-based HRP reaction product.. J Histochem Cytochem.

[PDF_00612] Chin J. L., Banerjee D., Kadhim S. A., Kontozoglou T. E., Chauvin P. J., Cherian M. G. (1993). Metallothionein in testicular germ cell tumors and drug resistance. Clinical correlation.. Cancer.

[PDF_00615] Chiu R., Boyle W. J., Meek J., Smeal T., Hunter T., Karin M. (1988). The c-Fos protein interacts with c-Jun/AP-1 to stimulate transcription of AP-1 responsive genes.. Cell.

[PDF_00618] Fresno M., Wu W., Rodriguez J. M., Nadji M. (1993). Localization of metallothionein in breast carcinomas. An immunohistochemical study.. Virchows Arch A Pathol Anat Histopathol.

[PDF_00621] Goulding H., Jasani B., Pereira H., Reid A., Galea M., Bell J. A., Elston C. W., Robertson J. F., Blamey R. W., Nicholson R. A. (1995). Metallothionein expression in human breast cancer.. Br J Cancer.

[PDF_00626] Haga A., Nagase H., Kito H., Sato T. (1996). Effect of metallothioneins on transformation of gelatinase A from human fibroblast WI-38 cells.. Cancer Lett.

[PDF_00624] Haga A., Nagase H., Kito H., Sato T. (1996). Enhanced invasiveness of tumour cells after host exposure to heavy metals.. Eur J Cancer.

[PDF_00629] Hall P. A., Levison D. A., Woods A. L., Yu C. C., Kellock D. B., Watkins J. A., Barnes D. M., Gillett C. E., Camplejohn R., Dover R. (1990). Proliferating cell nuclear antigen (PCNA) immunolocalization in paraffin sections: an index of cell proliferation with evidence of deregulated expression in some neoplasms.. J Pathol.

[PDF_00634] Hickey K., Grehan D., Reid I. M., O'Briain S., Walsh T. N., Hennessy T. P. (1994). Expression of epidermal growth factor receptor and proliferating cell nuclear antigen predicts response of esophageal squamous cell carcinoma to chemoradiotherapy.. Cancer.

[PDF_00638] Hishikawa Y., Abe S., Kinugasa S., Yoshimura H., Monden N., Igarashi M., Tachibana M., Nagasue N. (1997). Overexpression of metallothionein correlates with chemoresistance to cisplatin and prognosis in esophageal cancer.. Oncology.

[PDF_00642] Hollstein M. C., Metcalf R. A., Welsh J. A., Montesano R., Harris C. C. (1990). Frequent mutation of the p53 gene in human esophageal cancer.. Proc Natl Acad Sci U S A.

[PDF_00645] Hu E., Mueller E., Oliviero S., Papaioannou V. E., Johnson R., Spiegelman B. M. (1994). Targeted disruption of the c-fos gene demonstrates c-fos-dependent and -independent pathways for gene expression stimulated by growth factors or oncogenes.. EMBO J.

[PDF_00649] Jain S., Filipe M. I., Hall P. A., Waseem N., Lane D. P., Levison D. A. (1991). Prognostic value of proliferating cell nuclear antigen in gastric carcinoma.. J Clin Pathol.

[PDF_00654] Jasani B., Elmes M. E. (1991). Immunohistochemical detection of metallothionein.. Methods Enzymol.

[PDF_00652] Jasani B., Schmid K. W. (1997). Significance of metallothionein overexpression in human tumours.. Histopathology.

[PDF_00661] Karin M., Eddy R. L., Henry W. M., Haley L. L., Byers M. G., Shows T. B. (1984). Human metallothionein genes are clustered on chromosome 16.. Proc Natl Acad Sci U S A.

[PDF_00658] Karin M., Richards R. I. (1982). Human metallothionein genes--primary structure of the metallothionein-II gene and a related processed gene.. Nature.

[PDF_00664] Kasahara K., Fujiwara Y., Nishio K., Ohmori T., Sugimoto Y., Komiya K., Matsuda T., Saijo N. (1991). Metallothionein content correlates with the sensitivity of human small cell lung cancer cell lines to cisplatin.. Cancer Res.

[PDF_00667] Kelley S. L., Basu A., Teicher B. A., Hacker M. P., Hamer D. H., Lazo J. S. (1988). Overexpression of metallothionein confers resistance to anticancer drugs.. Science.

[PDF_00670] Kinugasa S., Tachibana M., Hishikawa Y., Abe S., Yoshimura H., Monden N., Dhar D. K., Nagasue N. (1996). Prognostic significance of proliferating cell nuclear antigen (PCNA) in squamous cell carcinoma of the esophagus.. Jpn J Clin Oncol.

[PDF_00674] Kitagawa Y., Ueda M., Ando N., Shinozawa Y., Shimizu N., Abe O. (1991). Significance of int-2/hst-1 coamplification as a prognostic factor in patients with esophageal squamous carcinoma.. Cancer Res.

[PDF_00677] Koji T., Brenner R. M. (1993). Localization of estrogen receptor messenger ribonucleic acid in rhesus monkey uterus by nonradioactive in situ hybridization with digoxigenin-labeled oligodeoxynucleotides.. Endocrinology.

[PDF_00681] Koji T., Nakane P. K. (1996). Recent advances in molecular histochemical techniques: in situ hybridization and southwestern histochemistry.. J Electron Microsc (Tokyo).

[PDF_00684] Kondo Y., Kuo S. M., Watkins S. C., Lazo J. S. (1995). Metallothionein localization and cisplatin resistance in human hormone-independent prostatic tumor cell lines.. Cancer Res.

[PDF_00687] Le Beau M. M., Diaz M. O., Karin M., Rowley J. D. (1985). Metallothionein gene cluster is split by chromosome 16 rearrangements in myelomonocytic leukaemia.. Nature.

[PDF_00690] Lee W., Mitchell P., Tjian R. (1987). Purified transcription factor AP-1 interacts with TPA-inducible enhancer elements.. Cell.

[PDF_00696] Mandard A. M., Marnay J., Gignoux M., Segol P., Blanc L., Ollivier J. M., Borel B., Mandard J. C. (1984). Cancer of the esophagus and associated lesions: detailed pathologic study of 100 esophagectomy specimens.. Hum Pathol.

[PDF_00699] Meltzer S. J., Yin J., Huang Y., McDaniel T. K., Newkirk C., Iseri O., Vogelstein B., Resau J. H. (1991). Reduction to homozygosity involving p53 in esophageal cancers demonstrated by the polymerase chain reaction.. Proc Natl Acad Sci U S A.

[PDF_00703] Miyake S., Nagai K., Yoshino K., Oto M., Endo M., Yuasa Y. (1994). Point mutations and allelic deletion of tumor suppressor gene DCC in human esophageal squamous cell carcinomas and their relation to metastasis.. Cancer Res.

[PDF_00707] Mulder T. P., Verspaget H. W., Janssens A. R., de Bruin P. A., Griffioen G., Lamers C. B. (1990). Neoplasia-related changes of two copper (Cu)/zinc (Zn) proteins in the human colon.. Free Radic Biol Med.

[PDF_00710] Nagel W. W., Vallee B. L. (1995). Cell cycle regulation of metallothionein in human colonic cancer cells.. Proc Natl Acad Sci U S A.

[PDF_00712] Ofner D., Maier H., Riedmann B., Bammer T., Rumer A., Winde G., Böcker W., Jasani B., Schmid K. W. (1994). Immunohistochemical metallothionein expression in colorectal adenocarcinoma: correlation with tumour stage and patient survival.. Virchows Arch.

[PDF_00716] Ohshio G., Imamura T., Okada N., Wang Z. H., Yamaki K., Kyogoku T., Suwa H., Yamabe H., Imamura M. (1996). Immunohistochemical study of metallothionein in pancreatic carcinomas.. J Cancer Res Clin Oncol.

[PDF_00721] Oyama T., Take H., Hikino T., Iino Y., Nakajima T. (1996). Immunohistochemical expression of metallothionein in invasive breast cancer in relation to proliferative activity, histology and prognosis.. Oncology.

[PDF_00724] Ransone L. J., Verma I. M. (1990). Nuclear proto-oncogenes fos and jun.. Annu Rev Cell Biol.

[PDF_00726] Schmid K. W., Ellis I. O., Gee J. M., Darke B. M., Lees W. E., Kay J., Cryer A., Stark J. M., Hittmair A., Ofner D. (1993). Presence and possible significance of immunocytochemically demonstrable metallothionein over-expression in primary invasive ductal carcinoma of the breast.. Virchows Arch A Pathol Anat Histopathol.

[PDF_00732] Schulte E. K., Lyon H. O., Hoyer P. E. (1992). Simultaneous quantification of DNA and RNA in tissue sections. A comparative analysis of the methyl green-pyronin technique with the gallocyanin chromalum and Feulgen procedures using image cytometry.. Histochem J.

[PDF_00740] Steele G. D., Davis S., Yow H., Wong J. M., Rivers E. N., Summerhayes I. C., Ravikumar T. S., Chen L. B. (1990). Alterations of gene expression in human colorectal cancer. Biological implications.. Arch Surg.

[PDF_00746] Tohyama C., Suzuki J. S., Hemelraad J., Nishimura N., Nishimura H. (1993). Induction of metallothionein and its localization in the nucleus of rat hepatocytes after partial hepatectomy.. Hepatology.

[PDF_00749] Yoshii A., Koji T., Ohsawa N., Nakane P. K. (1995). In situ localization of ribosomal RNAs is a reliable reference for hybridizable RNA in tissue sections.. J Histochem Cytochem.

[PDF_00752] Zelger B., Hittmair A., Schir M., Ofner C., Ofner D., Fritsch P. O., Böcker W., Jasani B., Schmid K. W. (1993). Immunohistochemically demonstrated metallothionein expression in malignant melanoma.. Histopathology.

[PDF_00762] Zheng H., Liu J., Choo K. H., Michalska A. E., Klaassen C. D. (1996). Metallothionein-I and -II knock-out mice are sensitive to cadmium-induced liver mRNA expression of c-jun and p53.. Toxicol Appl Pharmacol.

[PDF_00609] al-Sheneber I. F., Shibata H. R., Sampalis J., Jothy S. (1993). Prognostic significance of proliferating cell nuclear antigen expression in colorectal cancer.. Cancer.

